# Experimental-Numerical Failure Analysis of Thin-Walled Composite Columns Using Advanced Damage Models

**DOI:** 10.3390/ma14061506

**Published:** 2021-03-19

**Authors:** Patryk Rozylo, Katarzyna Falkowicz, Pawel Wysmulski, Hubert Debski, Jakub Pasnik, Jan Kral

**Affiliations:** 1 Department of Machine Design and Mechatronics, Faculty of Mechanical Engineering, Lublin University of Technology, Nadbystrzycka 36, 20-618 Lublin, Poland; k.falkowicz@pollub.pl (K.F.); p.wysmulski@pollub.pl (P.W.); h.debski@pollub.pl (H.D.); j.pasnik@pollub.pl (J.P.); 2 Faculty of Mechanical Engineering, Technical University of Kosice, Letna 9, 042 00 Kosice, Slovakia; kral.jan@tuke.sk

**Keywords:** finite element method (FEM), post-buckling, laminates, progressive failure analysis, cohesive zone model

## Abstract

The paper analyzes the stability and failure phenomenon of compressed thin-walled composite columns. Thin-walled columns (top-hat and channel section columns) were made of carbon fiber reinforced polymer (CFRP) composite material (using the autoclave technique). An experimental study on actual structures and numerical calculations on computational models using the finite element method was performed. During the experimental study, post-critical equilibrium paths were registered with acoustic emission signals, in order to register the damage phenomenon. Simultaneously to the experimental tests, numerical simulations were performed using progressive failure analysis (PFA) and cohesive zone model (CZM). A measurable effect of the conducted experimental-numerical research was the analysis of the failure phenomenon, both for the top-hat and channel section columns (including delamination phenomenon). The main objective of this study was to be able to evaluate the delamination phenomenon, with further analysis of this phenomenon. The results of the numerical tests showed a compatibility with experimental tests.

## 1. Introduction

Thin-walled composite structures are often used as stiffening elements in aviation, automotive, or construction industries. Both closed and open sections are used as load-carrying stiffening elements. Previously mentioned structures made of composite materials are mainly responsible for axial load-carrying. This type of structures should operate in the stable range (the increase in the post-critical deflection is directly accompanied by the load increase). The stability issue of this type of profiles is presented in many research papers [1,2,3]. Buckling can occur as a result of axial loading on thin-walled structures [4,5,6]. During the stable work [7,8,9,10,11], the structure is able to carry the axial compressive load after buckling [12,13]. Thin-walled composite structures (with flat walls) often have a high reserve of load-carrying capacity, especially after buckling [14,15,16]. In the stability analysis of structures, the post-buckling phase is very important. The phase mentioned above determines the behavior of the construction where increasing compressive load is accompanied by increase in buckling (in the case of stable work) [16,17,18]. The analysis of this type of structure is often associated with the possibility of damaging the composite structure [19,20,21,22,23]. When conducting research in the full load range, up to the structure’s failure, it is important to capture the failure phenomenon (both damage initiation and further damage evolution) [24,25,26,27]. First, Ply Failure theory states that the composite structure is damaged when the first layer of laminate is damaged [28]. Experimental tests enables to capture damage phenomenon with the use of acoustic emission signals [29]. Likewise, the numerical study allows capturing damage initiation when applying Hashin’s criterion [30]. After damage initiation phenomenon occurred, the damage evolution of the composite material begins. In Continuum Damage Mechanics (CDM), all types of damage are represented by the structural stiffness degradation [31,32]. The appropriate interpretation of the mentioned phenomenon defines the occurrence of certain micro-cracks or loss of the effective cross-sectional region [33]. For numerical analysis, the progressive failure analysis (PFA) requires the declaration of damage initiation and evolution parameters [34]. The progressive failure analysis is controlled by five variable damage components [35,36,37], responsible for: damage due to fibers compression or tension, damage due to matrix compression or tension and damage due to interlayer shearing [38,39,40]. For of complex interlayer behavior, e.g., delamination phenomenon, additional numerical techniques are used—based on cohesive zone model (CZM) [41,42]. The above-mentioned advanced model combines crack initiation criteria, based on stress components, with the energetic crack mechanics criteria [43,44]. The innovation in the present study is primarily the use of two independent damage models PFA and CZM (in each of the numerical models—both for top-hat and channel numerical models). The CZM model considered globally modeled cohesive surfaces, between all laminate layers (occurring on entire surfaces between layers)—which in most research studies are usually considered locally (i.e., only in regions where damage was observed in experimental studies). The subject literature has already described studies of the phenomenon of failure (including the phenomenon of delamination) of thin-walled composite materials. Those studies are mainly based on the example of columns with top-hat cross-section [45], which have higher load capacity and different geometrical parameters than those in the current research. This paper presents the phenomenon of failure in more details by comparing the failure results of top-hat and channel structures (which is the main difference in relation to previous research works). Current studies present a complex issue, with globally occurring cohesive zones between laminate layers and can be considered innovative in the context of delamination modeling [46,47,48,49,50,51]. In this work, both experimental and numerical studies of failure of the thin-walled composite profiles (top-hat [16,17,24] and channel section profile [25]) were carried out. In numerical calculations, the nonlinear Newton–Raphson incremental-iteration method [52], with parallel using damage criteria [53,54], was used. In case of experimental testing, a universal testing machine (UTM) and acoustic emission method (AEM) were used [55].

## 2. The Subject of the Study

**Figure 1:**
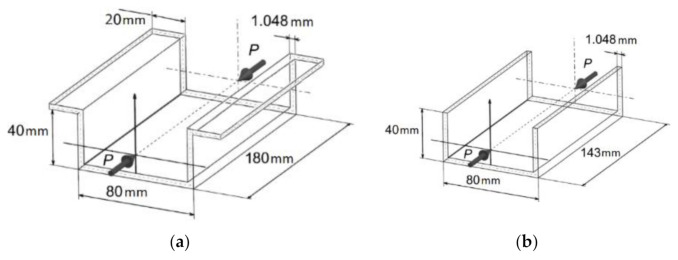
Geometrical parameters of specimens with layers arrangement: (**a**) top-hat; (**b**) channel.

The subject of the study was thin-walled top-hat and channel section composite structures made of CFRP material with the use of autoclave technique. The manufacturing process of specimens took place in a hermetic vacuum package in sterile conditions. The composite structure making method allowed for manufacture specimens with low porosity, high repeatability, and high strength properties [56]. The thin-walled composite columns were made of pre-preg (semi-finished components). The manufacturing process of composite specimens was presented in detail in the work [56]. The specimens consisted of eight layers (equal thickness) with symmetrical arrangement [45/–45/90/0]_s_ relative to the central laminate plane. The geometrical parameters of the specimens are shown in Figure 1.

Material properties (both mechanical and strength properties) were determined using a universal testing machine (UTM)—Zwick Z100 (ZwickRoell GmbH & Co. KG, Ulm, Germany). All mentioned properties were determined using ISO standards (ISO 14126—static compression test, ISO 527—static tensile test, ISO 14129—shear test) [45]. The mechanical and strength properties of the composite material are presented in Table 1 [56].

## 3. Experimental Investigation

**Figure 2:**
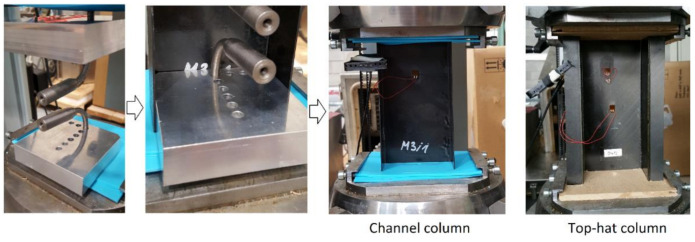
Experimental stand with mounted specimens.

In the presented work, the experimental study (EXP) of axial compression tests of thin-walled top-hat and channel section composite columns in full load range (up to loss of load-carrying capacity) were carried out. The axial compression was achieved with a constant cross-bar speed of 2 mm/min, at room temperature. In the experimental study, steel centering inserts were used in order to set the composite structure at the center of gravity. Additionally, special supports (plastic—for channel column, plywood—for top-hat column) were used in order to reduce any geometrical imperfections of end sections of actual specimens. Experimental tests were carried out using a UTM—Zwick Z100. The experimental study registered shortening, compressive force, and increase in deformation of the column (measured using the electrofusion strain gauges (Micro-Measurements, Raleigh, NC, USA), in the perpendicular direction to the web of composite structures, in the region of the largest deflections). Moreover, the acoustic emission method—AEM (registered: energy, amplitude, sum of hits, and number of counts) [29]—using the AMSY-5 device (Vallen Systeme GmbH, Icking, Germany) with special piezoelectric Fujicera sensor 1045S and AEP-4 signal amplifiers was used. An important objective of the conducted study was to determine post-critical equilibrium paths, which allows to predict the behavior of the composite columns subjected to axial compression. The test stand is shown in Figure 2.

During the experimental study, a variety of phenomena were recorded that directly contributed to the failure of the composite structures. As part of the study, the following issues were determined: damage initiation load—corresponding to the damage of the first laminate layer (*P*_d/EXP_), and the failure load (*P*_f/EXP_)—which corresponds to the loss of load-carrying capacity.

Regarding the above, the values of these loads (limit loads) were determined on the basis of the force-time characteristics. Additionally, selected acoustic emission signals were used to determine the values of the discussed loads.

## 4. Numerical Simulations

All numerical investigations were conducted using the finite element method (FEM) in ABAQUS^®^ software (Abaqus 2021, Dassault Systemes Simulia Corporation, Velizy Villacoublay, France). The numerical studies were conducted in two stages, with the first stage being solely the solution of the linear eigenproblem [57]—to determine the lowest buckling form corresponding to the experimental study.

The second stage of the numerical analysis dealt with a complex nonlinear stability problem of the structure including advanced numerical damage models. Numerical calculations were carried out using the Newton–Raphson method, considering amplitude of initial deflections (geometrical imperfections) *w*_0_ = 0.05 mm (which was determined in preliminary numerical calculations) [17].

The conducted simulation of structure damage was based on two independent damage models—progressive failure analysis and cohesive zone model.

The first damage model, known as progressive failure analysis (PFA), was based on the Hashin criterion (to describe damage initiation) [58,59]. Hashin’s damage initiation criterion includes four components of damage initiation: damage initiation due to fiber tension (HSNFTCRT) or compression (HSNFCCRT) and matrix tension (HSNMTCRT) or compression (HSNMCCRT). The fulfillment of any of the mentioned components determines the initiation of the damage initiation phenomenon of a given component (by obtaining a value of 1 within a given parameter), as well as possibility of damage evolution. Damage evolution requires introduction of the description of a special scalar damage parameter [33]. Scalar damage parameter can take values from 0 to 1 (0 indicates no damage evolution and 1 indicates damage to the structure due to the reach of evolution component). It can be conceptualized that the load is transferred solely through the undamaged cross-sectional structure region, which is represented by the effective stress. This assumption has been presented in many scientific papers [24,25,38]. When it comes to numerical analysis, it is possible to define the relationship between the nominal stress and effective stress using the damage operator *M*:

(1) $$\hat{\sigma }=M\sigma =\;\left[\begin{array}{ccc} \frac{1}{1-d_{f}} & 0 & 0\\ 0 & \frac{1}{1-d_{m}} & 0\\ 0 & 0 & \frac{1}{1-d_{s}} \end{array}\right]\left\{\begin{array}{c} \sigma_{11}\\ \sigma_{22}\\ \sigma_{12} \end{array}\right\}$$

where: σ denotes apparent stresses (Cauchy nominal), σ_ij_ defines stresses in the *ij* directions (σ_11_—local 1 direction corresponds to the fiber direction, σ_22_—local 2 direction corresponds to the direction perpendicular to the fiber, σ_12_—in the layer plane).

The variables shown in Relation (1), correspond consecutively:

(2) $$d_{f}=\{\begin{array}{c} d_{f}^{t},\;\;\;\;if\;\;\;{\hat{\sigma }}_{11}\geq 0,\\ d_{f}^{c},\;\;\;\;if\;\;\;{\hat{\sigma }}_{11}<0, \end{array}$$

(3) $$\;\;d_{m}=\{\begin{array}{c} d_{m}^{t},\;\;\;\;if\;\;\;{\hat{\sigma }}_{22}\geq 0,\\ d_{m}^{c},\;\;\;\;if\;\;\;{\hat{\sigma }}_{22}<0, \end{array}$$

(4) $$\;\;d_{s}=1-\left(1-d_{f}^{t}\right)\left(1-d_{f}^{c}\right)\left(1-d_{m}^{t}\right)\left(1-d_{m}^{c}\right)$$

The damage evolution model allows for a gradual stiffness degradation of the components [60], up to total loss of load-carrying capacity. The damage evolution phenomenon includes five parameters:$\;d_{f}^{t},\;d_{f}^{c}$, $d_{m}^{t}$, $d_{m}^{c}$, $d_{s}$, which correspond respectively: damage evolution due to fiber tension (DAMAGEFT), fiber compression (DAMAGEFC), matrix tension (DAMAGEMT), matrix compression (DAMAGEMC), and shear damage (DAMAGESHR).

**Figure 3:**
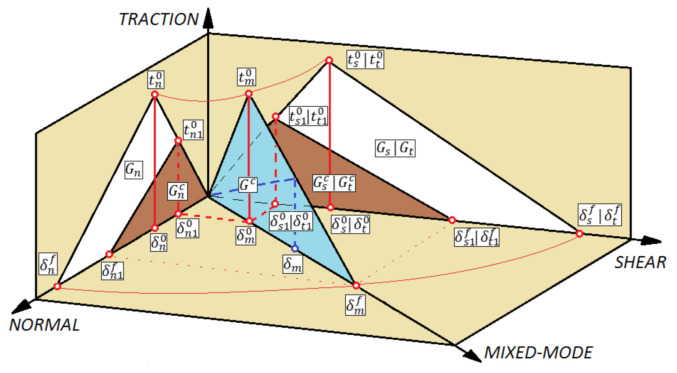
Traction-separation law (for cohesive model).

The second damage model—cohesive zone model (CZM)—enables the simulation of the delamination phenomenon of the composite structure (by rupture of the interlayer connections). The CZM model is based on traction-separation law (Figure 3) [61].

Regarding the elastic behavior of the cohesive layer, it is possible to represent this behavior by [62]:

(5) $$t=\left\{\begin{array}{c} t_{n}\\ t_{s}\\ t_{t} \end{array}\right\}=\left[\begin{array}{ccc} K_{nn} & K_{ns} & K_{nt}\\ K_{ns} & K_{ss} & K_{st}\\ K_{nt} & K_{st} & K_{tt} \end{array}\right]\left\{\begin{array}{c} \delta_{n}\\ \delta_{s}\\ \delta_{t} \end{array}\right\}=K\delta$$

where: *t, t_n_, t_s_, t_t_* denote cohesive tractions; *K, K_nn_, K_ss_, K_tt_* define the cohesive layer stiffness and δ, δ_n_, δ_s_, δ_t_ are the separation cohesive displacements, respectively, in global, normal, shear, and transverse shear directions.

The maximum nominal stress criterion (MAXS) has been used to determine the beginning of the cohesive degradation phenomenon. Regarding the MAXS criterion, when the maximum nominal stress obtains a value of 1 (parameter CSMAXSCRT obtain value of 1), damage process occurred [62]:

(6) $$max\left\{\frac{\langle t_{n}\rangle }{t_{n}^{0}},\;\frac{t_{s}}{t_{s}^{0}},\frac{t_{t}}{t_{t}^{0}}\right\}=1$$

where: $t_{n}^{0}$, $t_{s}^{0}$, $t_{t}^{0}$ define the peak values of the contact stress and 〈 〉 define a Macaulay bracket.

Regarding the CZM, a scalar damage variable (parameter *D*) represents overall damage (caused by delamination) in the composite material. In case of damage, the evolution of CZM scalar damage parameter monotonically evolves from value of 0 to 1 (after previously fulfilling the damage initiation). The parameter mentioned above can be presented as:

(7) $$D=\;\frac{\delta_{m}^{f}\left(\delta_{m}^{\max}-\delta_{m}^{0}\right)}{\delta_{m}^{\max}\left(\delta_{m}^{f}-\delta_{m}^{0}\right)}$$

where: $\delta_{m}^{0}$, $\delta_{m}^{f}$, $\delta_{m}^{\max}$ define respectively parameters of the effective displacement at damage initiation, effective displacement at total failure, and effective displacement at maximum value (obtained during the loading history).

The damage evolution, associated with the occurrence of progressive delamination phenomenon, is represented by the parameter CSDMG (which represents overall value of the scalar damage variable). The damage evolution of CZM is based on the energy criterion (B-K) [63]:

(8) $$G^{C}=\;G_{n}^{C}+\left(G_{s}^{C}-G_{n}^{C}\right){\left\{\frac{G_{S}}{G_{T}}\right\}}^{\eta }$$

where: *G^C^* defines the total critical fracture energy (in case of the mixed-mode behavior); $G_{n}^{C}$, $G_{s}^{C}$, $G_{t}^{C}$ define the critical fracture energies in normal, first and the second shear directions; *G_S_* denotes the portion of the total work (associated with shear traction and relative displacement); *G_T_* denotes total work (associated with normal and shear traction) and *η* denote a material parameter.

Similar to the PFA model, damage analysis is performed using the damage initiation and evolution phenomenon (based on energy criterion). 

**Figure 4:**
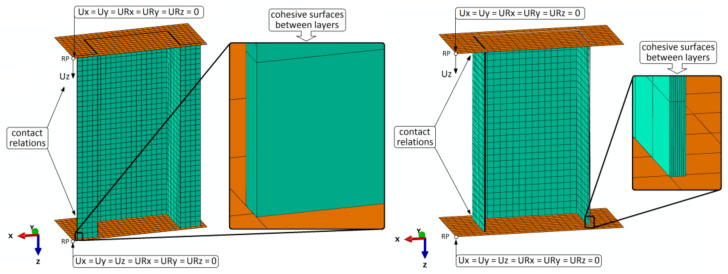
Discrete models of top-hat and channel columns with boundary conditions.

Regarding the prepared numerical models, a Continuum Shell model was used. All composite layers were modeled separately with each layer having a thickness of 0.131 mm. The orthotropic properties of each of the composite layers were declared. The numerical models were prepared using special type of finite elements (SC8R). Each of the finite elements had eight nodes (with three translational degrees of freedom, linear shape function and also reduced integration). Additionally, two non-deformable plates (representing supports of end sections of the composite structures) were modeled. The above-mentioned plates were designed using non-deformable Shell finite elements (R3D4). Each of the non-deformable finite elements have four computational nodes (containing three translational degrees of freedom). The discrete model of top-hat column had 13,296 finite elements and 27,342 nodes. For the discrete model of channel column, 8478 finite elements and 17,432 nodes were used. The reduced-density mesh used in the discrete models allowed numerical calculations to be performed without rapid loss of convergence as it is often the case with high-density mesh models. Regarding the numerical models, seven cohesive zones (cohesive surfaces) were implemented between eight laminate layers. The cohesive surfaces were introduced based on the parameters shown in Table 2. Additionally, between plates (constituting the supports for the end sections of the composite specimens) and the composite structure, the contact interactions were implemented—the tangential (with friction coefficient equal 0.2) and normal behavior. In the case of non-deformable plates, all boundary conditions were determined using reference points. The boundary conditions of the properly prepared numerical models are presented in Figure 4. A detailed description on the preparation of discrete models of thin-walled composite structures was presented in works on the subject literature [25,45].

The boundary conditions show high similarity between experimental and numerical studies; however, the lack of full symmetry is caused by the fact that the bottom head of the testing machine is fixed (like the bottom plate in numerical tests) and the top head moves in the direction of axial compression (like the top plate in numerical simulations). The additional influence on the asymmetrical results may be the lay-up system used, which, especially in the failure phase, can generate effects of locally occurring asymmetries in the damage regions. The un-symmetric damage evolution presented in the following section of the paper, was probably conditioned by the consideration of geometric imperfections at the level of *w*_0_ = 0.05 mm.

The damage initiation and evolution properties, associated with the PFA damage model [64] and CZM damage model [65] are presented in Table 2.

## 5. Results

**Figure 5:**
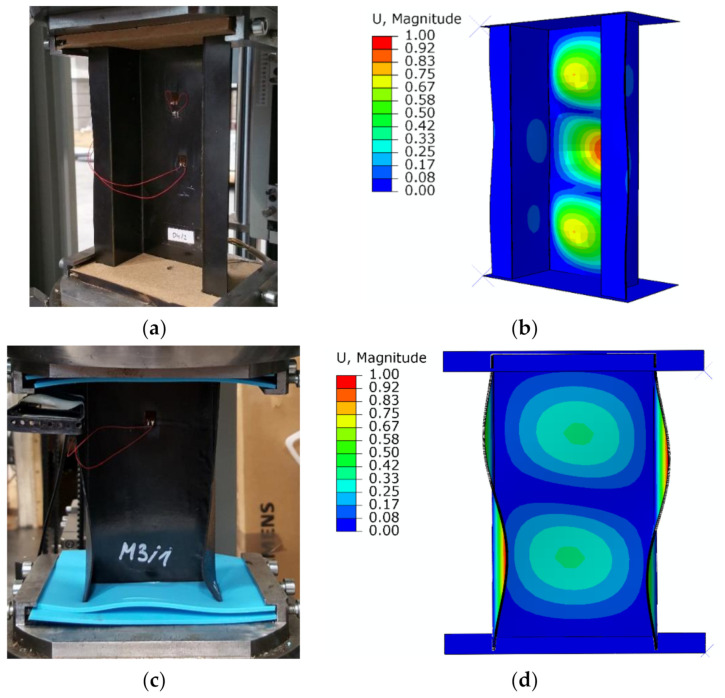
Buckling: (**a**) top-hat experimental study (EXP); (**b**) top-hat finite element method (FEM); (**c**) channel EXP; (**d**) channel FEM.

As part of the structures initially solved linear stability problem, a convergent result in the form of buckling were achieved. For the top-hat structure, three half-waves occurred in the longitudinal direction of the structure, while for the channel structure, two half-waves were obtained, as shown in Figure 5.

**Figure 6:**
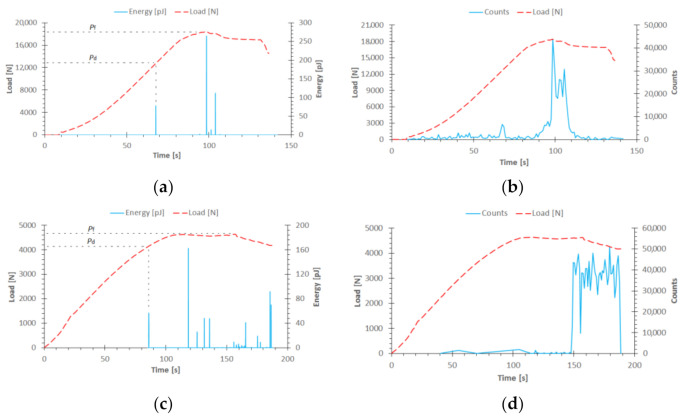
Post-critical equilibrium paths with acoustic emission signals: (**a**) top-hat (energy); (**b**) top-hat (counts); (**c**) channel (energy); (**d**) channel (counts).

The above result within the qualitative evaluation confirms the same behavior of the structure in experimental and numerical studies, in the initial loading range. The top-hat structure demonstrated higher stiffness (due to the two additional outer flanges) than the channel structure. Furthermore, as well as proper analysis of the behavior of the structure, the initiation and evolution of the damage can be evaluated. In the case of the experimental study, the damage initiation and the loss of load-carrying capacity were evaluated using the post-critical equilibrium paths *P-t* (load-time) compared with selected acoustic emission signals (energy and counts). The conducted experimental study demonstrated static (stable) behavior of structures (both for top-hat and channel columns). It also showed that the increase of the deflection is accompanied by the load increase [16,25], as shown in Figure 6.

**Figure 7:**
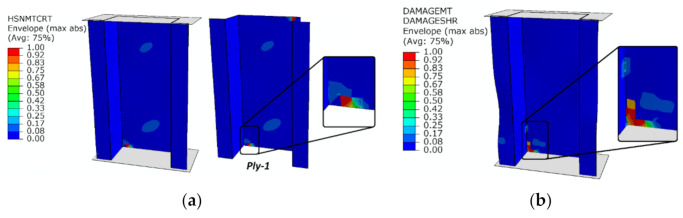

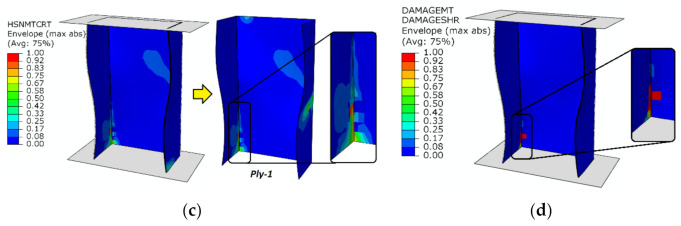
Damage initiation and evolution (PFA)—envelope result from eight laminate plies: (**a**) damage initiation of top-hat column—HSNMTCRT; (**b**) damage evolution of top-hat column—DAMAGEMT and DAMAGESHR; (**c**) damage initiation of channel column—HSNMTCRT; (**d**) damage evolution of channel column—DAMAGEMT and DAMAGESHR.

Based on the presented results for top-hat and channel columns, the damage initiation was directly evidenced by the first local observed “peak” of energy signal, which was approximately 75 pJ (at a time equal to 68 s) for top-hat column—Figure 6a, and 60 pJ (at a time equal to 85 s) for channel column—Figure 6c. A slight increase in the number of counts signal was also observed during the occurrence of the first energy “peak”. The acoustic emission signal presented in Figure 6b,d shows only the local increase in the signal (counts) in the time when the failure occurred and a significant increase in the signal was noted in its final phase—during the loss of the load capacity of the columns. The value of registered damage initiation load is *P*_d/EXP_ = 12,879.5 N for top-hat column and *P*_d/EXP_ = 4141.2 N for channel column. The value of the failure load was determined on the basis of the maximum value, which was registered during the experimental tests. During those tests a rapid increase in acoustic emission signals was recorded. The maximum registered load—failure load equaled *P*_f/EXP_ = 18,450.1 N for top-hat column and *P*_f/EXP_ = 4631.17 N for channel column. Regarding the experimental study, the damage initiation and failure loads for the top-hat column were a few times higher than for the channel column: 3.11 times higher in the context of damage initiation load and 3.98 times higher in the aspect of load causing loss of load-carrying capacity. During the parallel numerical simulations damage initiation and evolution were carried out with two damage models (PFA, CZM). In the first damage model, which was based on PFA, the damage initiation depended on Hashin criterion and damage evolution on energy criterion—Figure 7.

Obtaining a value of 1, indicating the fulfillment of the first component of damage initiation by HSNMTCRT, constituted the beginning of damage initiation. This occurred at a load that equals *P*_d/FEM-PFA_ = 13,567.66 N for top-hat column and *P*_d/FEM-PFA_ = 4303.36 N for channel column. The first layer to be damaged was the first external layer of the composite material (presented directly on Figure 7a,c). Further numerical analysis allowed for evaluation of the damage evolution phenomenon. Damage analysis in the context of damage evolution confirmed the progression of damage due to matrix tension which in the case of damage evolution corresponds to the DAMAGEMT parameter (at the same time the shear damage component DAMAGESHR was fulfilled). The damage evolution occurred at the load that equals 18,422.9 N for the top-hat column and 4370.93 N for the channel column. The fulfillment of the damage evolution within the first component was not the reason for the loss of load-carrying capacity of the composite structures.

**Figure 8:**
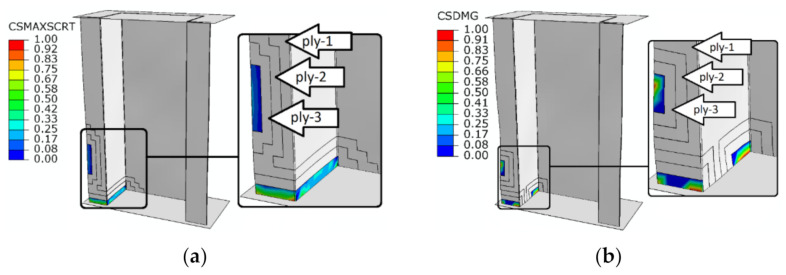

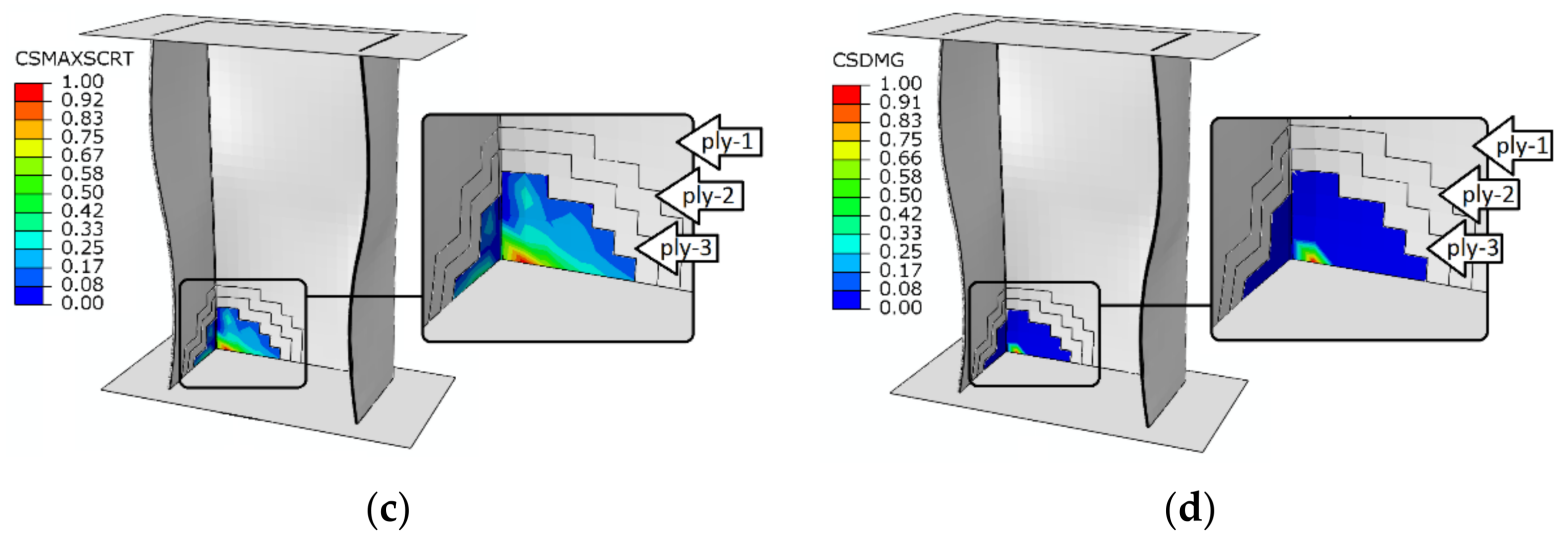
Damage initiation and evolution (CZM) caused by delamination: (**a**) damage initiation of top-hat column—CSMAXSCRT; (**b**) damage evolution of top-hat column—CSDMG; (**c**) damage initiation of channel column—CSMAXSCRT; (**d**) damage evolution of channel column—CSDMG.

For the second CZM damage model, damage initiation was only possible when fulfilling the MAXS, which is the maximum nominal stress criterion. The parameter responsible for achieving damage initiation was CSMAXSCRT. The considered parameter was fulfilled when a load value equaled *P*_d/FEM-CZM_ = 14,219.72 N for top-hat column and *P*_d/FEM-CZM_ = 4492.77 N for channel column. The damage initiation phenomenon due to delamination occurred between the third and fourth composite layers as shown in Figure 8. Damage analysis in the context of damage evolution confirmed the progression of damage due to delamination, which in the case of damage evolution corresponds to the CSDMG parameter. The damage evolution occurred at the load 19,080.53 N for top-hat column and 4612.79 N for channel column. In order to visualize the regions of damage initiation and evolution caused by delamination phenomenon, the first three plies of the composite columns were partially hidden. This allowed for better illustration of the damaged cohesive surface between the third and fourth plies of the composite structures—Figure 8.

As a part of the qualitative assessment, it was observed that damage initiation and damage evolution, regardless of the damage model used, occur mainly at the bottom parts of the end sections of the columns (both for top-hat and channel columns). In the case of both damage models (PFA and CZM), the progression of the damage evolution caused by further loading of the structures resulted in a complex damage mechanism—which directly contributed to the loss of load-carrying capacity of the structures. Loss of load-carrying capacity occurred at a load of *P*_f/FEM_ = 19,190.3 N for top-hat column and *P*_f/FEM_ = 4703.2 N for channel column. Regarding the numerical simulations, the damage initiation and failure loads for the top-hat column were a few times higher than for the channel column: 3.15 (based on PFA) and 3.17 (based on CZM) times higher in the case of damage initiation load, and 4.08 times higher in the aspect of load causing loss of load-carrying capacity. The numerical results were similar to the experimental results, where the top-hat column presented 3.11 times higher damage initiation load and 3.98 times higher failure load than the channel column. 

**Figure 9:**
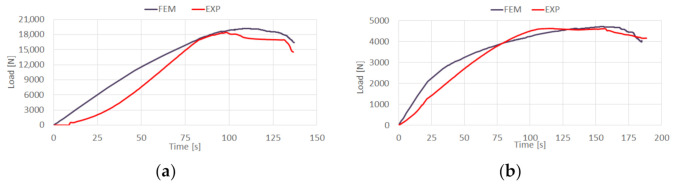
Comparison of post-critical equilibrium paths: (**a**) top-hat column, (**b**) channel column.

Additionally, in the context of the qualitative assessment, the experimental-numerical post-critical equilibrium paths were confronted—Figure 9.

**Figure 10:**
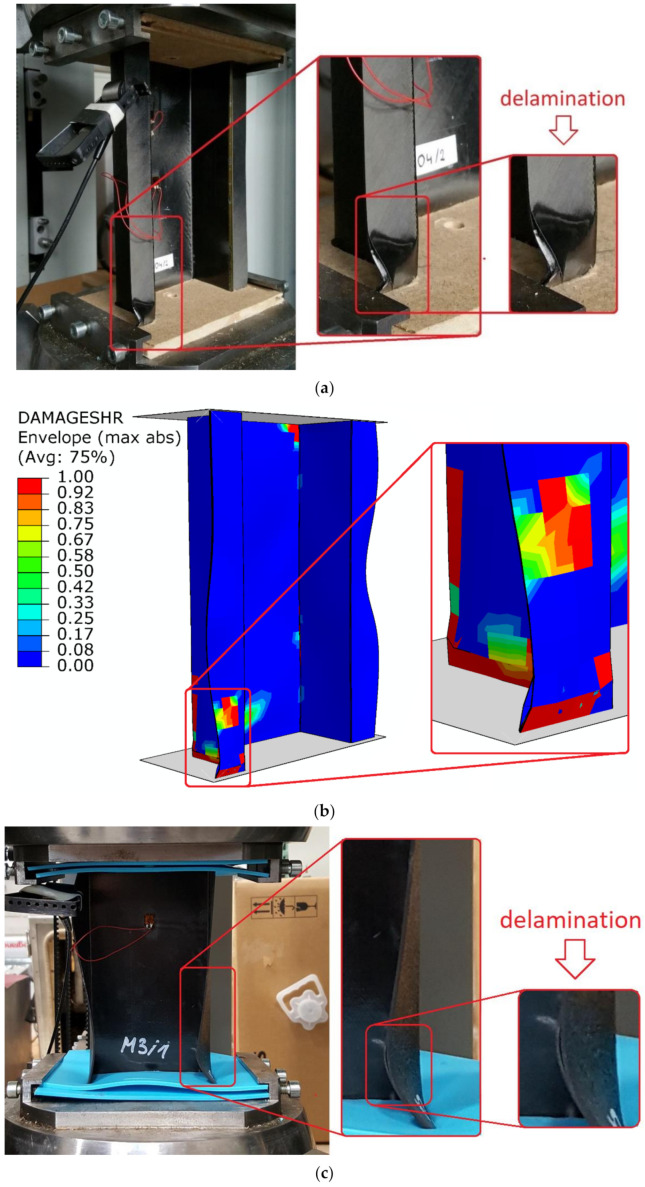

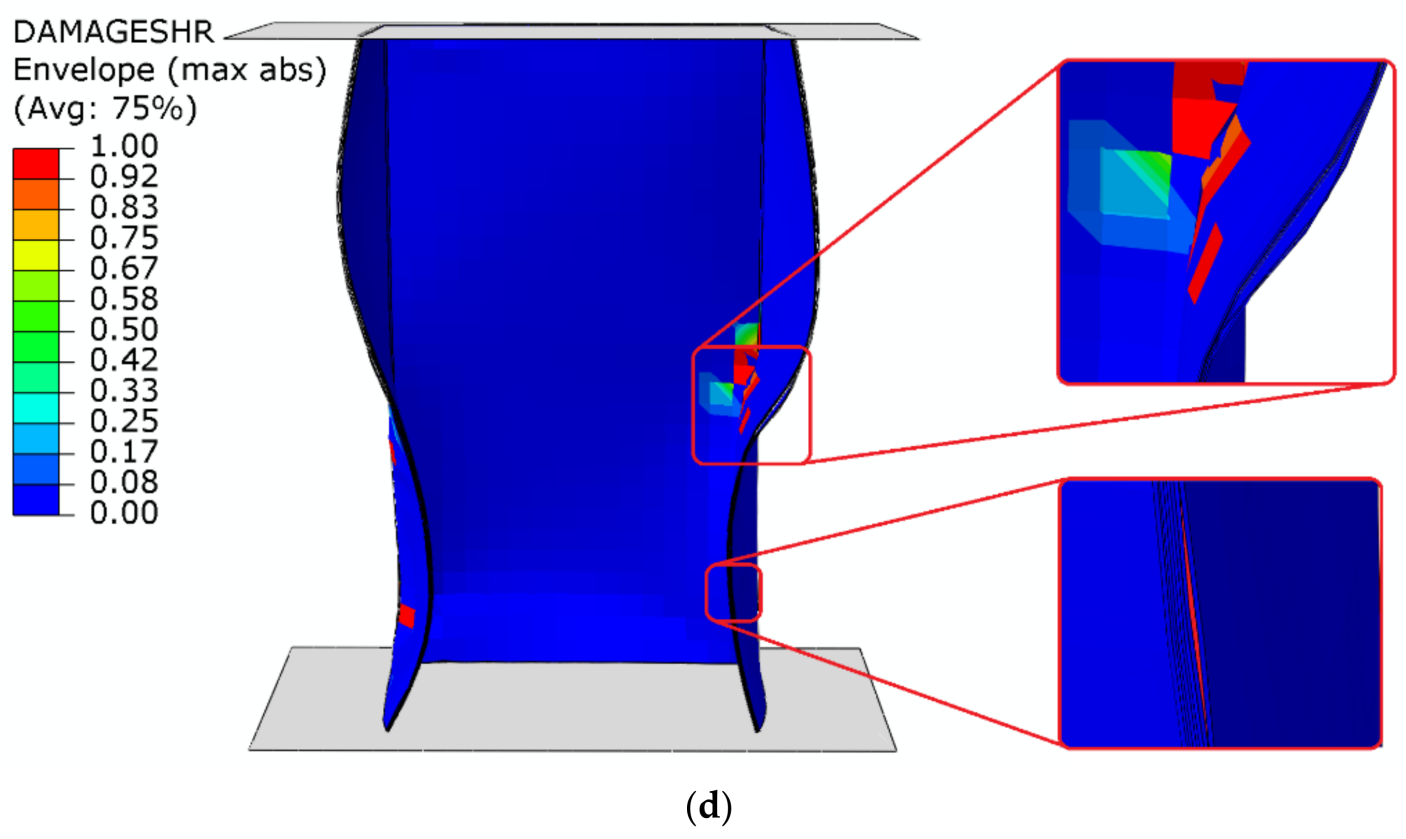
Qualitative assessment of loss of load-carrying capacity (including delamination): (**a**) top-hat column EXP; (**b**) top-hat column FEM; (**c**) channel column EXP; (**d**) channel column FEM.

Regarding the performed study, a high compliance between the experimental study and numerical simulations was observed [66,67,68,69,70,71,72,73,74]. Furthermore, the qualitative results of conducted investigations are presented in Figure 10 (including progressive delamination).

Despite the fact that the delamination phenomenon (according to Figure 8) was initiated in the bottom end sections of the columns—in the corner regions between the web and the flanges—a clear form of progressive damage caused by this phenomenon occurred mainly on one of the outer flanges (both for top-hat and channel column). Slight discrepancies between delamination results are conditioned by the fact that the numerical model represents an ideal construction and is not affected by any manufacturing imperfections (which is most often observed in case of actual specimens). Based on the results achieved, the loss of load-carrying capacity phenomenon (including delamination) of experimental and numerical tests showed highly similar results.

The quantitative evaluation was performed with the reference to the experimental and numerical tests of the failure analysis, as presented in Table 3.

**Figure 11:**
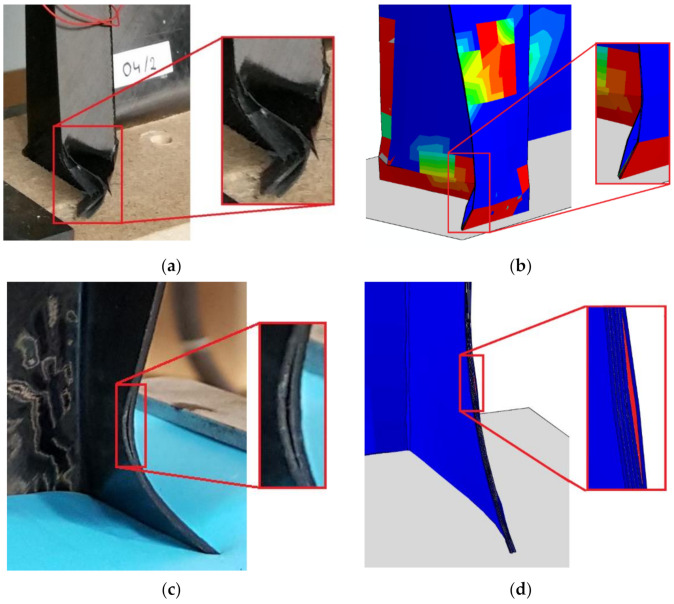
Comparison of delamination regions: (**a**) EXP (top-hat column); (**b**) FEM (top-hat column); (**c**) EXP (channel column); (**d**) FEM (channel column).

Additionally, the delamination regions in the detailed view is shown in Figure 11.

**Figure 12:**
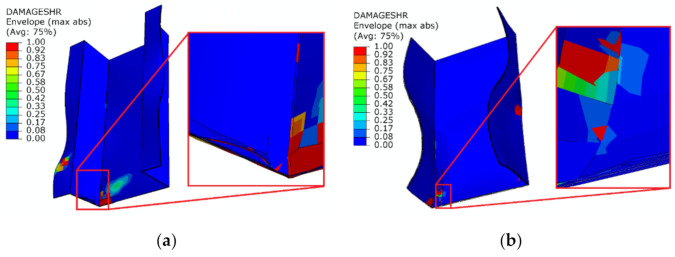
Additional delamination regions observed in numerical simulation: (**a**) top-hat column; (**b**) channel column.

In numerical studies, it was observed that delamination also occurred in regions where it would be difficult to estimate its occurrence experimentally, because this phenomenon occurred, inter alia, in the end sections—Figure 12.

The high agreement of result is conditioned by proper preparation of the numerical models (verified by experimental tests). Additionally, by comparing the obtained results for the top-hat column with the results presented in a previously published paper [45], it is possible to evaluate the influence of the stacking sequence on the load-carrying capacity of the composite structure. In the case of the structure presented in the previous publication [45], characterized by the lay-up [0/–45/45/90]_s_, two half-waves were observed in terms of the structure stability, and the failure load was equal to *P*_f_ = 17,084 N (FEM). Regarding the current work, with composite lay-up [45/–45/90/0]_s_, three half-waves were observed in terms of structure stability, and the failure load was equal to *P*_f_ = 19,190.3 N (FEM). The above proves the significant effect of stacking sequence (for composite layers) on the load carrying capacity of composite structures with the same shape but different lay-ups. The obtained results allowed for failure analysis of thin-walled composite structures with different cross-sections [75].

## 6. Conclusions

Regarding the thin-walled structures made of composite materials, it is important to conduct analyses in the post-critical state—considering the failure phenomenon. It is essential to evaluate the failure phenomenon in context of: damage initiation, delamination phenomenon, and also loss of load-carrying capacity. Therefore, it is necessary to conduct a study with use of independent experimental methods (universal testing machine, acoustic emission method) and a numerical model using advanced damage techniques (progressive failure analysis, cohesive zone model), in the full load range. Based on the conducted tests, the main conclusions are as follows:

- it is possible to evaluate the complex failure phenomenon of thin-walled composite structures with the use of experimental tests and numerical calculations;
- the evaluation of limit loads (damage initiation, damage evolution, beginning of delamination, loss of load-carrying capacity) allows to estimate the complexity of the failure phenomenon;
- for short composite columns, the delamination phenomenon and loss of load-carrying capacity propagate in the regions of end sections and outer flanges of the columns;
- composite structures with a top-hat cross section display a few times higher load-carrying capacity than structures with channel cross section;
- depending on the needs, it is possible to design profiles with higher stiffness—structures with a top-hat cross-section, as well as with lower stiffness—structures with a channel cross-section.

The research displayed that the current experimental and numerical research techniques allow for more in-depth analysis of the loss of load carrying capacity phenomenon (including delamination). The study exhibits an advanced modeling technique of the composite structures using advanced numerical damage models while considering traditional experimental methods. In the future, the study of stability and load carrying capacity of the structure will be carried out on several types of cross-sectional shapes of composite structures with consideration of the effect of different composite lay-ups on the behavior of the structure under axial compression. Additionally, an extended finite element method (XFEM) damage model will be used to simulate the initiation and propagation of material cracking.

## Figures and Tables

**Table 1:** The mechanical and strength properties of the composite material [56].

Mechanical Properties		Strength Properties	
Young’s modulus *E*_1_ [MPa]	130,710	Tensile Strength (0°) *F*_T1_ [MPa]	1867
Young’s modulus *E*_2_ [MPa]	6360	Compressive Strength (0°) *F*_C1_ [MPa]	1531
Poisson’s ratio	0.32	Tensile Strength (90°) *F*_T2_ [MPa]	26
Kirchhoff modulus *G*_12_, *G*_23_, *G*_13_ [MPa]	4180	Compressive Strength (90°) *F*_C2_ [MPa]	214
*-*	-	Shear Strength *F*_12_ [MPa]	100

**Table 2:** Progressive failure analysis (PFA) and cohesive zone model (CZM) parameters.

PFA Parameters								
Longitudinal tensile strength *X*^T^ [MPa]	Longitudinal compressive strength *X*^C^ [MPa]		Transverse tensile strength *Y*^T^ [MPa]	Transverse compressive strength *Y*^C^ [MPa]		Longitudinal shear strength *S*^L^ [MPa]		Transverse shear strength *S*^T^ [MPa]
1867	1531		26	214		100		107
Fracture energy *G*_1t_ fiber tension [N/mm]		Fracture energy *G*_1c_ fiber comp. [N/mm]	Fracture energy *G*_2t_ matrix crack. [N/mm]		Fracture energy *G*_2c_ matrix crush. [N/mm]		Viscosity coefficients η_1t_, η_1c_, η_2t_, η_2c_ [-]	
133		10	0.5		1.6		0.0005	
**CZM Parameters**								
Interface stiffness in normal, first and second shear direction *K*_nn_, *K*_ss_, *K*_tt_ [N/mm^3^]			Damage initiation stress in normal direction *t*_n_ [N/mm^2^]	Damage initiation stress in first and second shear direction *t*_s_, *t*_t_ [N/mm^2^]		Fracture energy in normal direction *G*_n_^C^ [N/mm]		Fracture energy in first and second shear direction *G*_s_^C^, *G*_t_^C^ [N/mm]
10^5^			18	14		0.32		0.68

**Table 3:** Results of damage initiation and failure loads.

Column Type	Limit Load	EXP [N]	FEM-PFA [N]	FEM-CZM [N]	EXP/FEM-PFA [%]	EXP/FEM-CZM [%]
Top-hat	Damage initiation (*P*_d_)	12,879.5	13,567.66	14,219.72	5.07	9.43
	Failure (*P*_f_)	18,450.1	19,190.3		3.86	
Channel	Damage initiation (*P*_d_)	4141.2	4303.36	4492.77	3.77	7.83
	Failure (*P*_f_)	4631.17	4703.2		1.53	

## Data Availability

Data is contained within the article.
